# Trends and burden of gout among adolescents aged 10–24 years: insights from the global burden of disease study 2021

**DOI:** 10.3389/fpubh.2025.1526141

**Published:** 2025-06-04

**Authors:** Yueshan Pang, Yiran Yuan, Wei Zhou, Shan Liu, Yanbin Du

**Affiliations:** Department of General Practice, Beijing Anzhen Nanchong Hospital of Capital Medical University & Nanchong Central Hospital, Sichuan, China

**Keywords:** gout, GBD, adolescent, trend, prediction

## Abstract

**Background:**

Gout increasingly affects adolescents. However, comprehensive data on adolescent gout are scarce. This study aimed to analyse the incidence, prevalence, years lived with disability (YLDs), and risk factors for gout in adolescents aged 10–24 years via the Global Burden of Disease (GBD) database from 1990 to 2021.

**Methods:**

This study employed cross-sectional data from the GBD 2021 study in adolescents aged 10–24 years. The incidence, prevalence, YLD, and risk factor rates or numbers for gout were extracted, and trends across different sociodemographic index (SDI) levels and exponential annualized percentage change (EAPC) rates were analysed via linear regression analysis. Bayesian age-period-cohot (BAPC) model with an integrated nested Laplace approximation to predict the age-standardized incidence rate of gout among adolescents over the next 15 years in Global, China, Taiwan (Province of China), Qatar, and Maldives.

**Results:**

The global incidence number of gout in adolescents increased by 23.47% (95% CI 21.66 to 25.92) from 1990 to 2021; by 2021, the incidence number of gout was 110664.59 (95% UI 62543.83 to 163447.37). High BMI was the leading risk factor for YLD. The higher the SDI is, the greater the incidence of gout and the greater the disease burden in adolescents. Taiwan (Province of China) had the highest rates in 2021, and an increasing trend is predicted by 2035.

**Conclusion:**

Adolescent gout is an escalating global health issue, particularly in regions with increasing age-standardized incidence rates. Urgent dietary and weight interventions are recommended to reduce the disease burden in adolescents.

## Introduction

Gout is a type of crystalline inflammatory arthritis characterized by the accumulation of monosodium urate crystals within joint structures, leading to chronic conditions. Studies have reported an increasing incidence of gout among adolescents (1). Gout not only causes joint pain and deformities in adolescents (2) but also leads to liver and kidney dysfunction, dyslipidemia (2, 3), and hypertension (2), promoting the progression of chronic kidney disease (CKD) and triggering cardiovascular diseases (2). Additionally, it increases the risk of degenerative neurological disorders (4), diminishes adolescents’ interpersonal skills and stress coping abilities (5), and even increases the risk of mortality (2, 6). Therefore, the epidemic of gout among adolescents requires attention from various countries and regions; however, there have been no reports on the prevalence of adolescent gout in the recent Global Burden of Disease Study 2021 (GBD 2021).

The incidence of gout has increased in young people. Zhang et al. (1) reported a significant increase in the gout incidence among adolescents aged 15–39 years in 2019 compared with 1990. From 1990 to 1999, the incidence of gout was 12 cases per 255,950 males and 1 case per 246,346 females under the age of 25 in Britain (7). There is also report related to adolescent gout in Korea (8). From 2007 to 2015, in South Korea, the incidence of gout among individuals aged 0 to 9 years was reported to be 2 to 3 cases per 100,000 people; however, in the 10 to 19 age group, the incidence was 9 to 20 cases per 100,000 people (8). In 2021, the global incidence of gout increased by 22.5% compared with that in 1990 (9). However, there have been no reported cases of gout among adolescents aged 10–24 years in the Global Burden of Disease (GBD) 2021.

This study utilizes the GBD database to analyse trends in incidence, prevalence, years lived with disability (YLDs) related to gout, and risk factors among adolescents aged 10–24 years from 1990 to 2021. Additionally, an attribution analysis of the countries or regions with the most significant increase in incidence is conducted, and their prevalence trends are predicted. This study will promote global, regional, and national attention to the prevalence of gout among adolescents. These findings will contribute to the development of new prevention strategies and treatment approaches worldwide, helping to alleviate the burden of gout in this age group and improve health outcomes.

## Methods

### Study population and data collection

This study utilized repeated cross-sectional data obtained from the GBD (2021), which assessed the burden of 371 diseases and 88 risk factors in 21 regions and 204 countries and territories from 1990 to 2021^1^ (10, 11). The GBD project is led by the Institute for Health Metrics and Evaluation at the University of Washington, which works collaboratively with partners to collect and curate the comprehensive dataset. The GBD 2021 data have been standardized, including input data, age and sex segmentation, cause of death clustering and noise reduction; then, the main models, such as the cause of death ensemble model, spatiotemporal Gaussian process regression and DisMod-MR, have been used for analysis (11).

The incidence of gout tends to occur at a relatively young age (1). This study adopts the broad age definition of 10–24 years old for adolescents, as it accurately captures the biological, social, and neurocognitive development of this population (12). Using GBD 2021, we extracted the following data, including incidence, prevalence, years lived with disability, and risk factors for gout among adolescents aged 10–24 years and conducted a secondary analysis. This study involved only data analysis and no identifiable personal information.

We conducted an analysis of the disease burden of gout among adolescents across 21 global regions, 204 countries and territories, and different regions at the sociodemographic index (SDI) level. The SDI has a scale ranging from 0 to 1, where higher values signify greater development of the social economy. The five quintiles of low, low-middle, middle, high-middle, and high were divided according to the different SDI levels. We analysed the relationships between different SDI levels across countries and regions and gout among adolescents.

### Statistical analysis

The incidence, prevalence, and YLDs of gout in adolescents were extracted from the GBD 2021, along with their corresponding age-standardized rates, as the main indicators used to describe the disease burden of gout in adolescents. In the GBD database, the age-standardized rate is estimated using the world population age standard. The direct standardization method yields standardized rates (also termed age-adjusted rates), which represent weighted averages of age-specific rates. These weights, derived from the relative age distribution of an external reference population, are applied uniformly across study populations to compute a summary measure. This approach estimates the expected disease burden under the assumption that all compared populations share identical age structures, thereby enabling cross-population comparisons while controlling for demographic variations. According to the GBD algorithms, a 95% uncertainty interval (UI) is provided for every rate reported per 100,000 people. The estimated annual percentage change (EAPC) was used to evaluate the temporal trend between 1990 and 2021. If both the EAPC and its upper 95% confidence interval (CI) were negative, the age-standardized rate exhibited a downward trend; accordingly, if both the EAPC and its lower limit of the 95% CI were greater than 0, the age-standardized rate exhibited a an increasing trend. To conduct the prediction analysis, we used the Bayesian age-period-cohort (BAPC) model on the basis of the literature (13). The BAPC model is a statistical model that considers the influence of age, period, and cohort factors on disease distribution. It combines the sample information and prior information through the Bayesian method to obtain unique parameter estimates, which makes the results more robust and reliable. BAPC models can not only describe the trends of diseases according to age, period, and birth cohort but also predict future changes on the basis of these trends. Analysis of the incidence, prevalence, and YLD of gout among adolescents from 1990 to 2021 was conducted via joinpoint regression to identify temporal trends.

All the data were statistically analyzed via GraphPad Prism (version 8.0), the Joinpoint Regression Program (version 5.0.2), and R (version 4.2.3). *p* < 0.05 was considered statistically significant.

## Results

### Global trends

#### Incidence

The incidence of gout among adolescents aged 10–24 years globally increased from 89630.16 (95% UI 50708.96 to 133466.20) in 1990 to 110664.59 (95% UI 62543.83 to 163447.37) in 2021, with a percentage change (PC) of 23.47% (95% UI 21.66 to 25.92). The age-standardized incidence rate increased from 5.79 per 100,000 people (95% UI 3.28 to 8.63) in 1990 to 5.86 per 100,000 people (95% UI 3.31 to 8.66) in 2021, with an EAPC of 0.39 (95% CI 0.21 to 0.56) from 1990 to 2021 (Table 1).

**Table 1:** Incidence of gout in 10–24 years old between 1990 and 2021 at the global and regional level.

Location	Rate per 100,000 (95% UI)					
	1990 (95% UI)		2021 (95% UI)		1990–2021	
	Incidence cases	Age-standardized incidence rates	Incidence cases	Age-standardized incidence rates	Cases change (%) (95% UI)	Rates EAPC (%) (95% CI)
Global	89630.16 (50708.96–133466.20)	5.79 (3.28–8.63)	110664.59 (62543.83–163447.37)	5.86 (3.31–8.66)	23.47 (21.66–25.92)	0.39 (0.21–0.56)
High	15251.63 (8834.97–22091.92)	7.79 (4.51–11.28)	19851.10 (11989.41–27842.28)	10.70 (6.46–15.00)	30.16 (20.60–43.82)	1.51 (1.30–1.71)
High middle	19811.65 (11343.40–29210.33)	6.98 (4.00–10.29)	16811.29 (9729.10–24499.96)	7.44 (4.31–10.85)	−15.14 (−18.14 – −11.80)	0.86 (0.53–1.19)
Middle SDI	34871.45 (19906.56–51134.56)	6.35 (3.63–9.32)	34620.06 (19777.25–51378.11)	6.26 (3.58–9.30)	−0.72 (−2.87 – −1.17)	0.32 (0.10–0.54)
Low middle	14165.27 (7734.54–21944.70)	3.92 (2.14–6.07)	25239.67 (13909.27–38640.83)	4.57 (2.52–6.99)	78.18 (73.29–85.08)	0.62 (0.57–0.66)
Low SDI	5469.36 (2894.45–8559.10)	3.51 (1.86–5.50)	14064.40 (7670.11–21822.36)	3.81 (2.08–5.91)	157.15 (151.36–167.23)	0.24 (0.21–0.27)
Regions						
Andean Latin America	494.31 (258.83–778.23)	4.02 (2.10–6.32)	995.64 (544.71–1520.70)	5.77 (3.16–8.81)	101.42 (81.32–128.53)	1.22 (1.19–1.25)
Australasia	363.57 (225.00–527.14)	7.56 (4.68–10.96)	600.14 (350.45–867.37)	10.46 (6.11–15.12)	65.07 (47.05–94.17)	1.20 (1.05–1.34)
Caribbean	450.10 (234.24–707.47)	4.21 (2.19–6.63)	584.48 (318.12–905.88)	5.16 (2.81–8.00)	29.85 (22.00–42.34)	0.75 (0.63–0.87)
Central Asia	809.92 (437.02–1245.86)	4.08 (2.20–6.28)	1026.08 (564.81–1566.49)	4.64 (2.55–7.08)	26.69 (19.00–35.75)	1.01 (0.78–1.23)
Central Europe	1008.74 (515.21–1602.00)	3.45 (1.76–5.49)	738.39 (393.59–1167.76)	4.07 (2.17–6.44)	−26.80 (−30.44 – −21.93)	0.70 (0.51–0.88)
Central Latin America	2131.21 (1114.91–3438.74)	3.93 (2.05–6.34)	3350.93 (1838.30–5302.46)	5.15 (2.83–8.15)	57.23 (48.02–70.21)	0.54 (0.39–0.68)
Central Sub-Saharan Africa	649.07 (350.45–1009.92)	3.75 (2.02–5.84)	1704.05 (902.08–2672.74)	3.79 (2.01–5.95)	162.54 (139.31–186.71)	0.10 (0.05–0.14)
East Asia	34668.93 (20697.02–49650.54)	9.32 (5.56–13.34)	22908.87 (13663.27–32620.73)	9.43 (5.62–13.42)	−33.92 (−36.46 – −31.35)	0.80 (0.38–1.23)
Eastern Europe	1887.25 (1000.96–2970.95)	4.00 (2.12–6.29)	1353.45 (736.33–2105.19)	4.10 (2.23–6.38)	−28.28 (−31.59 – −24.62)	0.73 (0.32–1.14)
Eastern Sub-Saharan Africa	2131.74 (1128.99–3379.84)	3.44 (1.82–5.45)	5576.84 (3022.95–8634.66)	3.83 (2.08–5.94)	161.61 (152.44–174.72)	0.32 (0.29–0.36)
High-income Asia Pacific	2521.14 (1444.30–3729.64)	5.98 (3.43–8.85)	1871.42 (1077.01–2731.85)	7.17 (4.13–10.46)	−25.77 (−29.59 – −21.00)	0.26 (0.14–0.38)
High-income North America	7220.23 (4267.75–10166.95)	11.80 (6.98–16.62)	11769.64 (7609.74–16045.64)	16.51 (10.68–22.51)	63.01 (47.44–88.50)	1.78 (1.49–2.08)
North Africa and Middle East	4408.48 (2425.91–6845.88)	4.05 (2.23–6.29)	7985.88 (4368.93–12204.24)	4.92 (2.69–7.52)	81.15 (71.39–92.62)	0.82 (0.69–0.95)
Oceania	141.82 (85.45–209.60)	6.78 (4.08–10.02)	304.34 (181.91–445.03)	7.55 (4.51–11.04)	114.59 (97.49–131.76)	0.35 (0.29–0.40)
South Asia	12277.91 (6481.92–19228.76)	3.67 (1.94–5.75)	22116.65 (11929.70–34681.90)	4.21 (2.27–6.60)	80.13 (75.31–85.83)	0.57 (0.50–0.63)
Southeast Asia	8755.59 (5066.74–12906.36)	5.90 (3.42–8.70)	12771.59 (7491.00–18805.09)	7.47 (4.38–11.00)	45.87 (39.34–54.45)	0.86 (0.83–0.90)
Southern Latin America	827.25 (471.29–1230.27)	6.25 (3.56–9.29)	1297.02 (749.49–1887.13)	8.46 (4.89–12.30)	56.79 (41.90–75.63)	0.95 (0.90–1.01)
Southern Sub-Saharan Africa	734.51 (402.42–1122.44)	4.30 (2.36–6.57)	1065.28 (598.82–1599.47)	4.88 (2.75–7.33)	45.03 (39.00–52.57)	0.65 (0.53–0.78)
Tropical Latin America	1718.61 (882.44–2759.19)	3.59 (1.84–5.77)	2535.06 (1363.71–3826.45)	5.01 (2.70–7.57)	47.51 (36.29–65.92)	1.20 (1.12–1.27)
Western Europe	4270.93 (2404.50–6537.35)	5.20 (2.93–7.95)	3888.32 (2172.30–5860.48)	5.39 (3.01–8.13)	−8.96 (−13.88 – −4.03)	0.14 (0.09–0.19)
Western Sub-Saharan Africa	2158.85 (1151.08–3384.27)	3.61 (1.92–5.66)	6220.54 (3381.40–9644.18)	3.85 (2.10–5.98)	188.14 (179.75–198.20)	0.19 (0.12–0.25)

#### Prevalence

The prevalence of gout among adolescents aged 10–24 worldwide increased from 179232.87 (95% UI 93890.46 to 296682.18) in 1990 to 220881.48 (95% UI 116542.40 to 364750.66) in 2021, with a PC 23.24% (95% UI 21.55 to 25.75). The age-standardized prevalence rate increased from 11.58 per 100,000 people (95% UI 6.07 to 19.18) in 1990 to 11.70 per 100,000 people (95% UI 6.17 to 19.32) in 2021, with an EAPC of 0.381 (95% CI 0.205 to 0.558) from 1990 to 2021 (Supplementary Table 1).

#### YLDs

Globally, the YLD for gout among adolescents aged 10–24 years increased from 6287.14 (95% UI 2958.23 to 10998.57) in 1990 to 7722.32 (95% UI 3648.10 to 13409.75) cases in 2021, with a PC of 22.83% (95% UI 19.88 to 26.45). The age-standardized YLD rate for gout among adolescents remained consistent between 1990 and 2021, with an EAPC of 0.368 (95% UI 0.19 to 0.54) from 1990 to 2021 (Supplementary Table 2).

### SDI regional trends

#### Incidence

**Figure 1:**
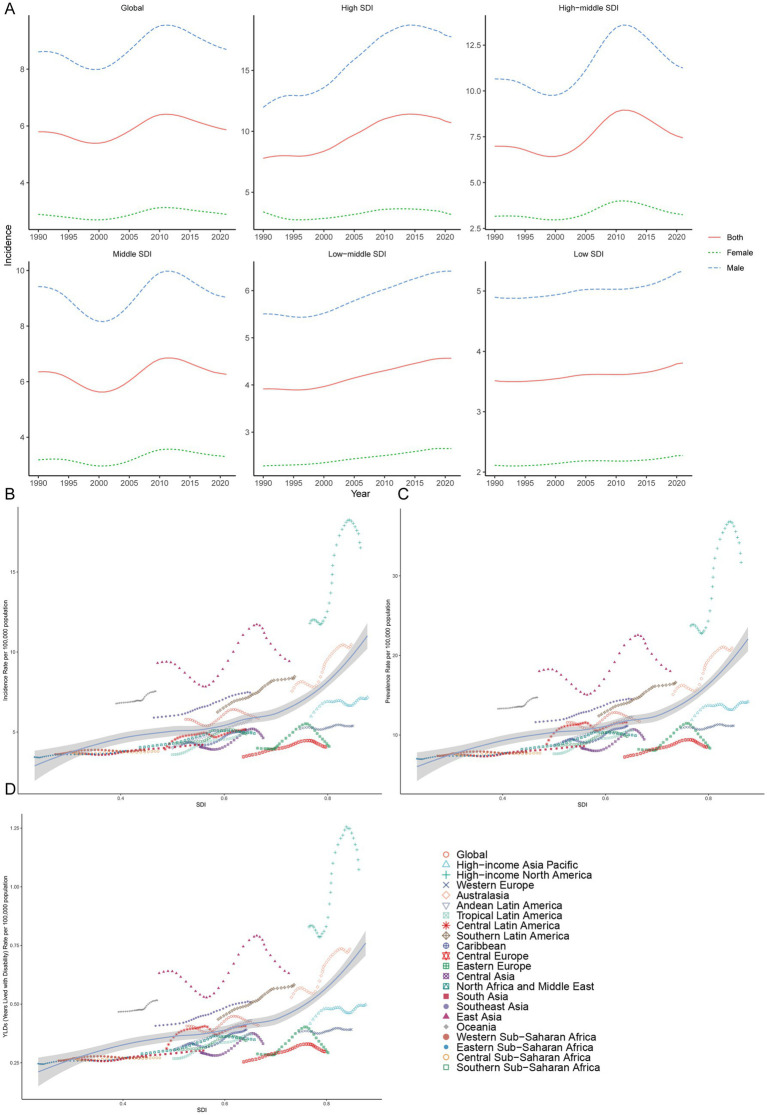
Prevalence of gout among adolescents in different SDI regions. **(A)** The age-standardized incidence rate of gout among adolescents in high, high-middle, middle, low-middle, low SDI regions. Incidence **(B)**, prevalence **(C)** and YDL **(D)** rates across in geographic regions.

The higher the SDI region is, the greater the incidence in adolescents. In regions with high, high-middle, and middle SDI, the age-standardized incidence rates of gout among adolescents peaked at 11.42 (95% UI 8.14 to 14.90), 8.95 (95% UI 5.26 to 13.05), and 6.85 (95% UI 3.92 to 10.13) per 100,000 people in 2014, 2011 and 2012, respectively, and then declined. Although the gout age-standardized incidence rate among adolescents was relatively low in low-middle and low SDI regions in 1990, there was a consistent increasing trend from 1990 to 2021. By 2021, the age-standardized incidence rate among adolescents had risen to 4.57 per 100,000 people (95% UI 2.52 to 6.99) in low-middle SDI regions and 3.81 per 100,000 people (95% UI 2.08 to 5.91) in low SDI regions. Regardless of the SDI, females presented a lower age-standardized incidence rate than males did (Table 1; Figure 1A).

#### Prevalence

In regions with high, high-middle, and middle SDI, the gout age-standardized prevalence rate among adolescents peaked at 23.06 (95% UI 15.53 to 32.85), 17.56 (95% UI 9.76 to 28.14) and 13.62 (95% UI 7.12 to 22.43) per 100,000 people in 2014, 2011 and 2012 respectively, and then declined. The gout age-standardized prevalence rate among adolescents was relatively low in low-middle and low SDI regions in 1990, but there was a consistent increasing trend from 1990 to 2021. By 2021, the age-standardized prevalence rates among adolescents had risen to 9.31 (95% UI 4.53 to 15.76) and 7.75 (95% UI 3.59 to 13.28) per 100,000 people in low-middle and low SDI regions, respectively. Regardless of the SDI, males had a higher age-standardized prevalence rate than females did among adolescents (Supplementary Table 1; Supplementary Figure 1).

#### YLDs

In regions with high, high-middle and middle SDI, the number of gout YLDs among adolescents peaked at 1530.14 (95% UI 874.97 to 2421.19), 1624.91 (95% UI 803.45 to 2809.99) and 2784.92 (95% UI 1343.24 to 4869.23) in 2013, 2010 and 2010 respectively, and then declined. The YLD among adolescents was relatively low in the low-middle and low SDI regions in 1990, but there was a consistent increasing trend from 1990 to 2021. By 2021, the YLDs had risen to 1805.84 (95% UI 794.88 to 3271.24) and 1005.11 (95% UI 441.51 to 1847.31) in the low-middle and low SDI regions, respectively. Regardless of the SDI, the gouty YLD among adolescents is greater in males than in females (Supplementary Figure 1B; Supplementary Table 2).

#### 21 geographic regional trends

**Figure 2:**
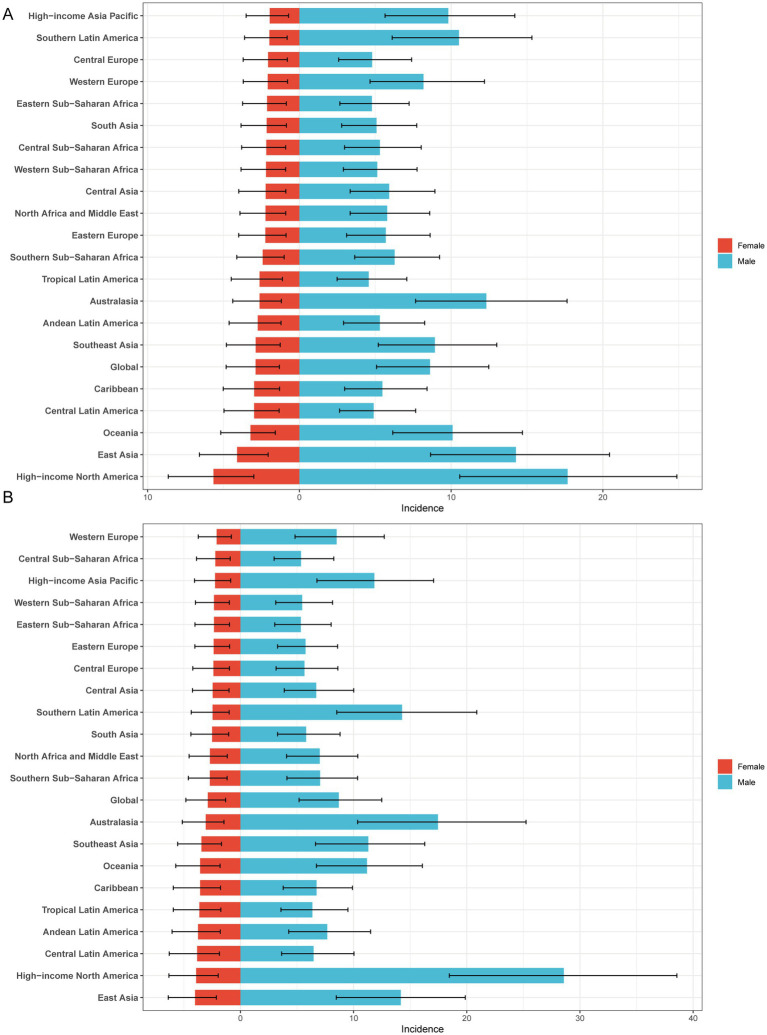
Sex-specific age-standardized incidence rate of gout in adolescents. **(A)** 1990 age-standardized incidence rate of gout in adolescents. **(B)** 2021 age-standardized incidence rate of gout in adolescents.

**Figure 3:**
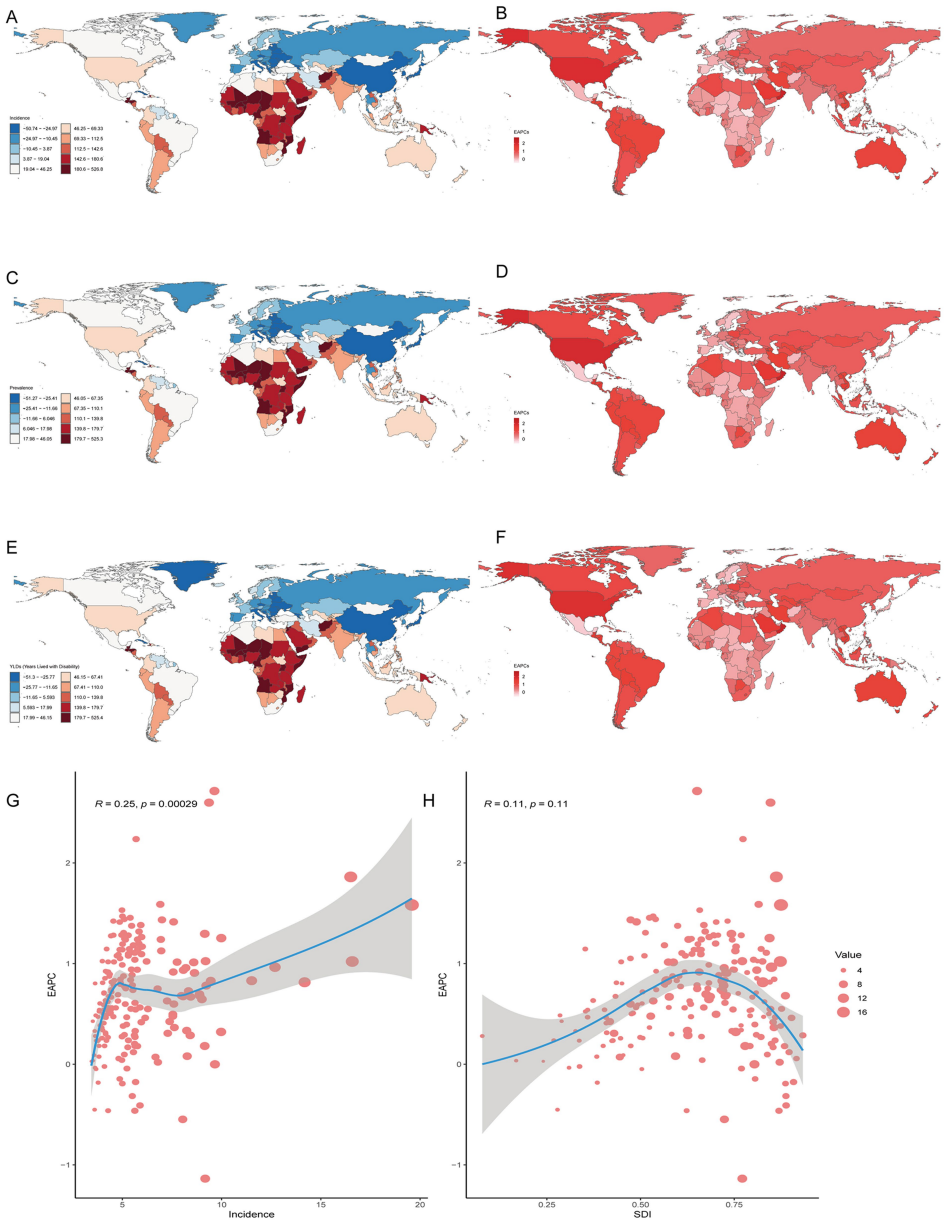
Prevalence of gout among adolescents in 204 countries and territories. **(A,C,E)** PCs of incidence, prevalence and YDL number. **(B,D,F)** EAPCs in the incidence, prevalence and YDL rates. **(G, H)** Analysis of correlations among incidence, EAPC, and SDI.

In both 1990 and 2021, among the 21 geographic regions, the regions with the highest incidence, prevalence and YLD number of gout cases among adolescents were East Asia; however, the highest age-standardized incidence, prevalence and YLD rates were found in high-income North America. The incidence of PC decreased 33.92% (95% UI-36.46 to-31.35) from 1990 to 2021 in East Asia. However, the age-standardized incidence rate of EAPC in high-income North America increased by 1.78 (95% CI 1.49 to 2.08). In both 1990 and 2021, Oceania had the lowest incidence, prevalence and YLD number of gout among adolescents, and central sub-Saharan Africa had the lowest age-standardized incidence, prevalence and YLD rates. In both 1990 and 2021, the age-standardized incidence rates of gout among adolescents were higher in males than in females across 21 geographic regions (Figures 2B–D, 3; Table 1; Supplementary Tables 1, 2). Our study found positive correlations between SDI and gout incidence, prevalence, and YLD rates across 21 regions. Higher SDI areas consistently showed elevated adolescent gout metrics, demonstrating a clear dose-response relationship (Figures 1B–D).

### National trends

In both 1990 and 2021, among the 204 countries and territories, the highest incidence, prevalence and number of YLDs among adolescents were in China. In 1990, the highest age-standardized incidence, prevalence and YLD rates of gout among adolescents were reported in Greenland. However, in 2021, the highest age-standardized incidence, prevalence and YLD rates were reported in Taiwan (Province of China). From 1990 to 2021, the greatest increase in the PC in the incidence, prevalence and YLD number of gout among adolescents was in Qatar, and the largest decrease was in Latvia. From 1990 to 2021, the greatest increase in the PC in the age-standardized incidence, prevalence and YLD rates of gout among adolescents occurred in Taiwan (Province of China), and the largest decrease occurred in the northern Mariana Islands. From 1990 to 2021, Romania exhibited the largest decrease in the incidence, prevalence and YLD number of EAPC, and Qatar exhibited the largest increase in the incidence, prevalence and YLD number of EAPC. From 1990 to 2021, the greatest increase in the age-standardized incidence, prevalence and YLD rates of EAPC was reported in Maldives, and the largest decrease was reported in the northern Mariana Islands. There was a linear relationship between the incidence, prevalence, YLD rate and EAPC; the higher the age-standardized incidence, prevalence and YLD rate were, the greater the EAPC was (*p* < 0.05). However, there was no significant correlation between the SDI and the incidence, prevalence, or YLD rates of EACP in 204 countries or territories (Table 2; Figure 3; Supplementary Figure 2).

**Table 2:** The top 1 nations or territories of incidence, prevalence, YLDs, PC and EAPC.

Burdens of gout among adolescents (case and rate)		1990 (location, value 95% UI)		2021 (location, value 95% UI)		1990–2021			
		Lowest	Most	Lowest	Most	Cases change (%) (location, value 95% UI)		Rate EAPC (%) (location, value 95% CI)	
						Decrease	Increase	Decrease	Increase
Incidence	cases	Tokelau, 0.03 (0.09–0.05)	China, 33470.93 (19972.80–47941.56)	Tokelau, 0.03 (0.09–0.04)	China, 21592.01 (12822.88–30840.12)	Latvia, −50.74 (−56.46 – −44.42)	Qatar, 526.83 (449.67–620.13)	Romania, −2.23 (−2.51 – −1.95)	Qatar, 9.08 (7.80–10.37)
	rates per 100,000	Yemen, 3.00 (1.49–4.86)	Greenland, 14.54 (8.59–20.85)	Chad, 3.44 (1.78–5.38)	Taiwan (Province of China), 19.59 (12.60–27.60)	Northern Mariana Islands, −11.26 (−22.87 – −1.44)	Taiwan (Province of China), 68.96 (50.10–97.83)	Northern Mariana Islands, −1.14 (−1.53 – −0.74)	Maldives, 2.71 (2.30–3.13)
Prevalence	cases	Tokelau, 0.06 (0.03–0.10)	China, 64691.25 (36666.85–103079.44)	Tokelau, 0.06 (0.03–0.09)	China, 41152.75 (23815.21–64186.99)	Latvia, −51.27 (−57.03 – −44.51)	Qatar, 525.30 (457.17–628.31)	Romania, −2.27 (−2.56–−1.971)	Qatar, 9.08 (7.80–10.37)
	rates per 100,000	Yemen, 6.10 (2.66–10.46)	Greenland, 29.95 (17.736–45.45)	Chad, 6.99 (3.07–11.93)	Taiwan (Province of China), 40.46 (24.50–61.82)	Northern Mariana Islands, −12.84 (−22.58 – −4.57)	Taiwan (Province of China), 76.16 (53.64–109.39)	Northern Mariana Islands, −1.27 (−1.67 – −0.86)	Maldives, 2.69 (2.26–3.11)
YLDs	cases	Tokelau, 0.00 (0.00–0.01)	China, 2269.78 (1140.512–3912.66)	Tokelau, 0.00 (0.00–0.00)	China, 1443.98 (743.35–2464.09)	Latvia, −51.30 (−57.03 – −44.51)	Qatar, 525.39 (457.17–628.31)	Romania, −2.26 (−2.55 – −1.97)	Qatar, 9.06 (7.79–10.36)
	rates per 100,000	Yemen, 0.21 (0.09–0.41)	Greenland, 1.05 (0.54–1.74)	Chad, 0.25 (0.11–0.47)	Taiwan (Province of China), 1.39 (0.41–2.81)	Northern Mariana Islands, −12.79 (−22.58 – −4.57)	Taiwan (Province of China), 72.24 (−28.93–194.22)	Northern Mariana Islands, −1.27 (−1.67 – −0.86)	Maldives, 2.68 (2.26–3.11)

#### Risk factors

**Figure 4:**
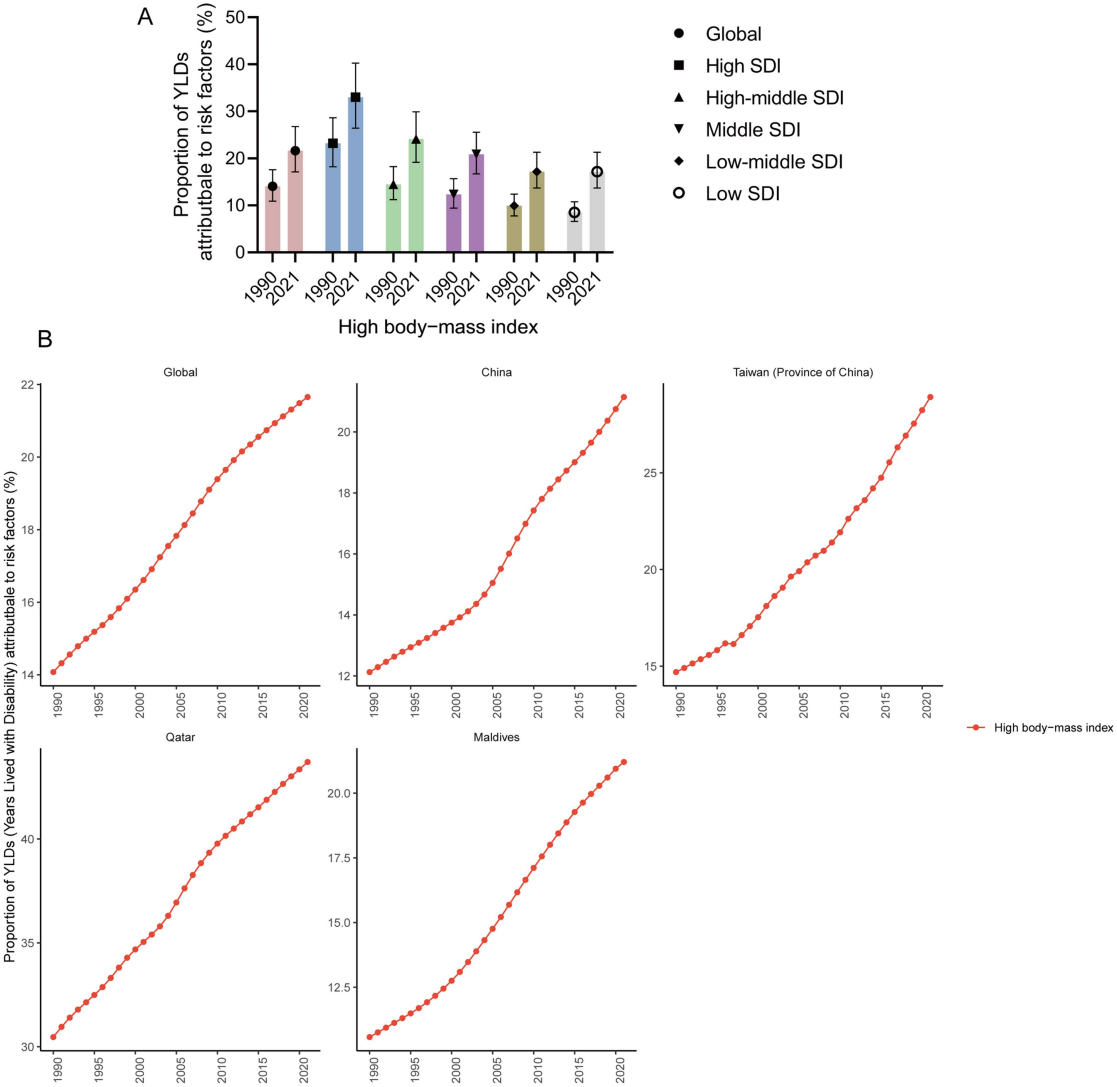
Proportion of YLDs attributable to risk factors. **(A)** Proportion of YLD attributable to risk factors in high, high-middle, middle, low-middle, and low SDI regions in 1990 and 2021 (95% UI). **(B)** Proportion of YLDs attributable to risk factors in global, China, Taiwan (Province of China), Qatar, and Maldives from 1990 to 2021.

For adolescents aged 10–24 years, a high BMI is the primary risk factor for YLD of gout among adolescents. Globally, in 2021, a high BMI accounted for 21.66% of YLDs (95% UI 17.14 to 26.76%). The higher the SDI is, the greater the impact of high BMI on YLD. In 2021, in high SDI regions, a high BMI accounted for 33.00% of YLDs (95% UI 26.43 to 40.25%). However, in low SDI regions, a high BMI accounted for only 13.09% of YLD (95% UI 10.39 to 16.21%). From 1990 to 2021, the proportion of YLDs attributed to a high BMI among adolescent gout patients increased, and there was a continuing increasing trend in global, China, Taiwan (Province of China), Qatar and Maldives. In 2021, the highest proportion of high BMIs, accounting for 43.71% (95% UI 0.34.87 to 52.86%) of YLDs, was reported in Qatar, followed by Taiwan (Province of China), accounting for 28.92% (95% UI 20.83 to 37.17%) (Figure 4).

#### BAPC analysis

**Figure 5:**
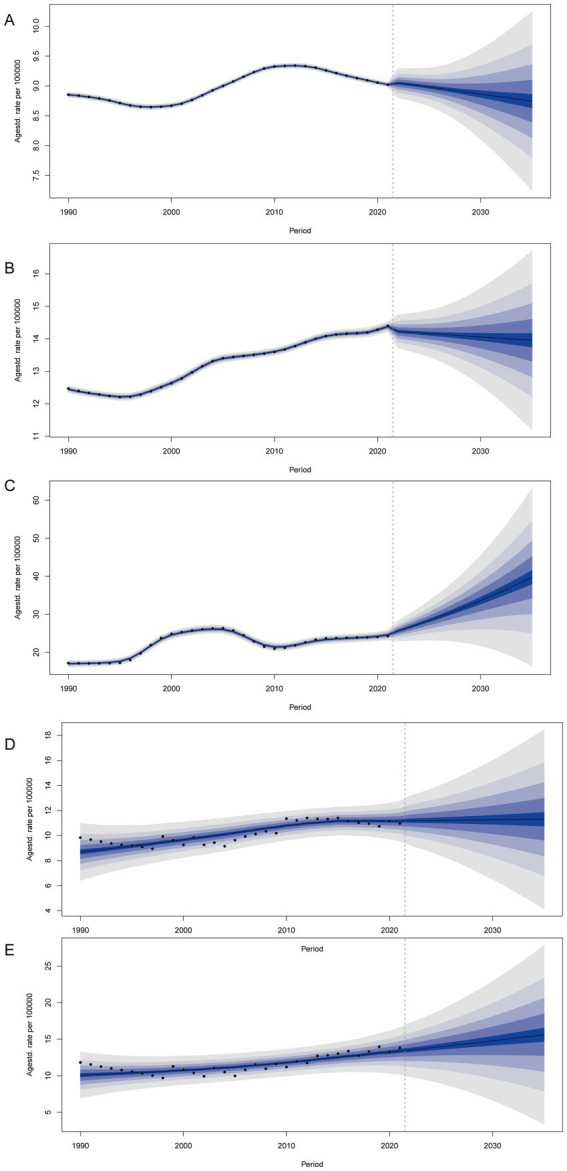
BAPC analysis of global **(A)**, China **(B)**, Taiwan (Province of China) **(C)**, Qatar **(D)**, and Maldives **(E)**.

We predicted the age-standardized incidence rate of gout among adolescents aged 10–24 years in global, China, Taiwan (Province of China), Qatar, and the Maldives. By 2035, it is projected that the age-standardized incidence rates of gout among adolescents will be on a declining trend both globally and in China. However, the age-standardized incidence rate of gout among adolescents in Taiwan (Province of China) and the Maldives still shows an increasing trend. By 2035, the incidence of gout among adolescents is expected to be 39.77 per 100,000 people (95% UI 11.85 to 67.69) in Taiwan (Province of China) and 15.61 per 100,000 people (95% UI 1.00 to 30.22) in the Maldives. By 2035, the age-standardized incidence rate of gout among adolescents in Qatar is not expected to change significantly (Figure 5).

## Discussion

Data from the GBD 2021 indicate an increase in the global incidence of gout (9); however, there have been no reports on the incidence of gout among adolescents aged 10–24 years. This is the first research report focusing on the incidence, prevalence, YLDs, risk factors, and predicted trends of gout in adolescents. From 1990 to 2021, there was a significant increase in the incidence, prevalence and YLD number and rates of gout among adolescents. A high BMI is the main risk factor for YLD. Gout can lead to various diseases in adolescents, such as joint deformities (2, 14), neurodegenerative changes (4), renal dysfunction (15), diabetes insipidus (16), and cardiovascular and cerebrovascular diseases (6). Adolescents are the reserve force of our social development. Therefore, focusing on adolescent health, reducing the disease burden of gout among adolescents, and improving health outcomes for adolescents are currently global public health issues.

The incidence of gout among adolescents, particularly males, has increased. In recent years, there have been frequent reports of gout cases among adolescents (17–19). There are many reasons why uric acid can be elevated in adolescents. Unhealthy eating habits are an important cause of gout (20). The consumption of excessive amounts of meat, seafood, carbonated beverages, and beer can increase blood uric acid levels (21–23). Genetic factors can increase the incidence of gout among adolescents. Mutations in SLC2A9 (24) and ABCG2 (25) can both lead to hyperuricemia and the occurrence of gout. Less physical activity were associated with a less favorable metabolic profile in adolescents (26). Less exercise easily leads to obesity. Obesity not only affects aesthetics but also, more importantly, increases the production and excretion of uric acid in the body, thereby increasing the risk of gout (27). The incidence of some underlying diseases, such as type 1 diabetes mellitus (28) and kidney disease (29), is increasing in adolescents, which increases the incidence of gout. Hormones are also a factor in gout (18). During puberty, high levels of testosterone in boys can increase serum uric acid levels, whereas estrogen in females can increase the excretion of uric acid (30). In summary, many causes lead to gout in adolescents. The prevention and control of gout in adolescents aged 10–24 years should be strengthened.

In 204 countries and territories, there was no significant correlation between the EAPC and the SDI. Changes in the EAPC may be related to policy. For example, the incidence of gout among Chinese adolescents has increased significantly with economic growth since 2001, reaching a peak in 2011 before beginning to decline. With the growth of China’s economy, the continuous improvement in the Chinese health level, and the implementation of a comprehensive medical security system, there is a certain relationship between these factors and the reduction in the incidence of gout (31).

High BMI is the primary risk factor for YLD, and it shows an increasing trend. This suggests that we should pay more attention to weight issues in the prevention and control of gout in adolescents. Excessive consumption of high-energy foods in adolescents is one of the reasons for high BMI and high uric acid levels (21, 32). High triglyceride levels can also increase the incidence of gout; for each unit increase in the triglyceride index, the risk of gout increases by 40% (33).

The prevalence of gout among adolescents shows a national or territorial trend. This study revealed that Taiwan (Province of China) had the highest age-standardized incidence, prevalence and YLD rate of gout among adolescents in 2021. Furthermore, an increasing trend in the age-standardized incidence rate was predicted by 2035. As early as 2003, a study reported that local children had relatively high levels of blood uric acid in Taiwan (Province of China) (34). Genetic factors have been implicated in the high prevalence of gout in Taiwan (Province of China) (35). The genes ABCG2 and SLC2A9 were found to be the major genetic factors governing gout in Taiwan (Province of China) (36). The prevalence of obesity in Taiwanese adolescents is another reason for the increased incidence of gout (37). Studies have shown that Taiwanese adolescents consume high amounts of fast food, high-fat snacks, processed meats, and sugar-sweetened beverages (38), which can increase the risk of gout. These findings suggest that more measures need to be taken in Taiwan (Province of China) to strengthen the prevention and control of gout among adolescents.

Strategies for coping with adolescent gout. The most direct method to control gout is to reduce and manage blood uric acid levels (39). Patients with blood uric acid levels ≤6.0 mg/dL have a lower incidence of gout (39). On the basis of the study (40), healthy eating guidelines or gout dietary guidelines for adolescents can be developed and publicized in the community or school. Exercise interventions should be carried out in adolescents to reduce the prevalence of obesity and risk factors for gout (41). For example, relevant policies can be developed to strengthen the assessment of physical activity among adolescents. For adolescents with a high BMI, losing weight is the most direct and safe way to reduce gout attacks (42, 43). Finally, for adolescents with familial inheritance tendencies, uric acid screening should be strengthened, and interventions related to diet, lifestyle, exercise and other aspects should be carried out as soon as possible.

The data for this study were obtained from the GBD database. Owing to differences in economic conditions across countries and regions, the data on adolescent gout in the GBD database are not comprehensive (9). In cases where data are insufficient to provide burden estimates for all 204 countries and territories, by year, sex, and age, data estimates depend on the out-of-sample predictive validity of the modeling process (11). The disease burden of gout in adolescents in low and middle economic areas may be much greater than that reported in the GBD database. Differences in healthcare systems, diagnostic standards, and availability of advanced diagnostic tools across regions can result in underdiagnosis or misclassification of gout, especially in areas with limited resources. For example, asymptomatic hyperuricemia or cases with unclear clinical presentations may not be consistently documented. Moreover, changes in diagnostic approaches over time can introduce temporal biases, making longitudinal comparisons more challenging. These inconsistencies may skew the accurate estimation of disease burden and highlight the need for careful interpretation when analyzing gout trends.

## Conclusion

Adolescent gout is a global public health issue, particularly in countries and regions where the age-standardized incidence rate is increasing rapidly. It is strongly recommended that adolescents’ dietary patterns be adjusted, weight intervention be strengthened, and the disease burden of gout in adolescents be reduced.

## Data Availability

The original contributions presented in the study are included in the article/Supplementary material, further inquiries can be directed to the corresponding author.

## Footnotes

^1^ https://vizhub.healthdata.org/gbd-results/

## References

[1] Zhang J Jin C Ma B Sun H Chen Y Zhong Y. Global, regional and national burdens of gout in the young population from 1990 to 2019: a population-based study. RMD Open. (2023) 9:e003025. doi: 10.1136/rmdopen-2023-003025, PMID: 37094981 PMC10152042

[2] Singh JA Gaffo A. Gout epidemiology and comorbidities. Semin Arthritis Rheum. (2020) 50:S11–6. doi: 10.1016/j.semarthrit.2020.04.008, PMID: 32620196

[3] Jia E Zhu H Geng H Liu R Wo X Zeng Y. The effects of aerobic exercise on body composition in overweight and obese patients with gout: a randomized, open-labeled, controlled trial. Trials. (2022) 23:745. doi: 10.1186/s13063-022-06695-x 36064594 PMC9446810

[4] Topiwala A Mankia K Bell S Webb A Ebmeier KP Howard I. Association of gout with brain reserve and vulnerability to neurodegenerative disease. Nat Commun. (2023) 14:2844. doi: 10.1038/s41467-023-38602-6, PMID: 37202397 PMC10195870

[5] Peng J Yin L Wang K Zhang T Liu H Yang J. A study on the relationship between adolescent health behavior, BMI, and blood physical and chemical properties. Front Public Health. (2022) 10:766101. doi: 10.3389/fpubh.2022.766101, PMID: 35372227 PMC8964522

[6] Ferguson LD Molenberghs G Verbeke G Rahimi K Rao S McInnes IB. Gout and incidence of 12 cardiovascular diseases: a case-control study including 152 663 individuals with gout and 709 981 matched controls. Lancet Rheumatol. (2024) 6:e156–67. doi: 10.1016/S2665-9913(23)00338-7, PMID: 38383089

[7] Mikuls TR Farrar JT Bilker WB Fernandes S Schumacher HR Jr Saag KG. Gout epidemiology: results from the UK general practice research database, 1990-1999. Ann Rheum Dis. (2005) 64:267–72. doi: 10.1136/ard.2004.024091, PMID: 15647434 PMC1755343

[8] Kim JW Kwak SG Lee H Kim SK Choe JY Park SH. Prevalence and incidence of gout in Korea: data from the national health claims database 2007-2015. Rheumatol Int. (2017) 37:1499–506. doi: 10.1007/s00296-017-3768-4, PMID: 28676911

[9] GBD 2021 Gout Collaborators. Global, regional, and national burden of gout, 1990-2020, and projections to 2050: a systematic analysis of the global burden of disease study 2021. Lancet. Rheumatol. (2024) 6:e507–17. doi: 10.1016/S2665-9913(24)00117-6, PMID: 38996590 PMC11263476

[10] GBD 2021 Risk Factors Collaborators. Global burden and strength of evidence for 88 risk factors in 204 countries and 811 subnational locations, 1990-2021: a systematic analysis for the global burden of disease study 2021. Lancet. (2024) 403:2162–203. doi: 10.1016/S0140-6736(24)00933-4, PMID: 38762324 PMC11120204

[11] GBD 2021 Diseases and Injuries Collaborators. Global incidence, prevalence, years lived with disability (YLDs), disability-adjusted life-years (DALYs), and healthy life expectancy (HALE) for 371 diseases and injuries in 204 countries and territories and 811 subnational locations, 1990-2021: a systematic analysis for the global burden of disease study 2021. Lancet. (2024) 403:2133–61. doi: 10.1016/S0140-6736(24)00757-8, PMID: 38642570 PMC11122111

[12] Sawyer SM Azzopardi PS Wickremarathne D Patton GC. The age of adolescence. Lancet Child Adolesc Health. (2018) 2:223–8. doi: 10.1016/S2352-4642(18)30022-1, PMID: 30169257

[13] Guo Y Zhang K Zou Y Zhang K Zou Y Yu B. National situation, trends, and predictions of disease burden of atopic dermatitis in Chinese children and adolescents. Front Microbiol. (2023) 14:1161969. doi: 10.3389/fmicb.2023.1161969, PMID: 37396371 PMC10308015

[14] Zhao J Sha B Zeng L Dou Y Huang H Liang G. J-shaped association of serum uric acid concentrations with all-cause mortality in individuals with osteoarthritis: a prospective cohort study. Joint Bone Spine. (2024) 91:105679. doi: 10.1016/j.jbspin.2023.105679, PMID: 38143017

[15] Venkat-Raman G Gast C Marinaki A Fairbanks L. From juvenile hyperuricaemia to dysfunctional uromodulin: an ongoing metamorphosis. Pediatr Nephrol. (2016) 31:2035–42. doi: 10.1007/s00467-015-3308-y, PMID: 26872483

[16] Zhang Y Wang D Feng Y Zhang W Zeng X. Juvenile-onset gout and adipsic diabetes insipidus: a case report and literature review. J Int Med Res. (2018) 46:4829–36. doi: 10.1177/0300060518800114, PMID: 30270804 PMC6259371

[17] Chen-Xu M Yokose C Rai SK Pillinger MH Choi HK. Contemporary prevalence of gout and hyperuricemia in the United States and decadal trends: the National Health and nutrition examination survey, 2007-2016. Arthritis Rheumatol. (2019) 71:991–9. doi: 10.1002/art.40807, PMID: 30618180 PMC6536335

[18] Ito S Torii T Nakajima A Iijima T Murano H Horiuchi H. Prevalence of gout and asymptomatic hyperuricemia in the pediatric population: a cross-sectional study of a Japanese health insurance database. BMC Pediatr. (2020) 20:481. doi: 10.1186/s12887-020-02379-0, PMID: 33059648 PMC7559194

[19] Kuo CF Grainge MJ See LC Yu KH Luo SF Valdes AM. Familial aggregation of gout and relative genetic and environmental contributions: a nationwide population study in Taiwan. Ann Rheum Dis. (2015) 74:369–74. doi: 10.1136/annrheumdis-2013-204067, PMID: 24265412 PMC4316854

[20] Alvarez-Lario B Alonso-Valdivielso JL. Hyperuricemia and gout; the role of diet. Nutr Hosp. (2014) 29:760–70. doi: 10.3305/nh.2014.29.4.7196 24679016

[21] Lu J Sun W Cui L Li X He Y Liu Z. A cross-sectional study on uric acid levels among Chinese adolescents. Pediatr Nephrol. (2020) 35:441–6. doi: 10.1007/s00467-019-04357-w, PMID: 31811538 PMC6968984

[22] Chen JX Yu XX Ye Y Yang XB Tan AH Xian XY. Association between recreational physical activity and the risk of upper urinary calculi. Urol Int. (2017) 98:403–10. doi: 10.1159/000452252, PMID: 27771724

[23] Zhou H Ma ZF Lu Y du Y Shao J Wang L. Elevated serum uric acid, hyperuricaemia and dietary patterns among adolescents in mainland China. J Pediatr Endocrinol Metab. (2020) 33:487–93. doi: 10.1515/jpem-2019-0265, PMID: 32069235

[24] Batt C Phipps-Green AJ Black MA Cadzow M Merriman ME Topless R. Sugar-sweetened beverage consumption: a risk factor for prevalent gout with SLC2A9 genotype-specific effects on serum urate and risk of gout. Ann Rheum Dis. (2014) 73:2101–6. doi: 10.1136/annrheumdis-2013-203600, PMID: 24026676 PMC4251167

[25] Toyoda Y Pavelcova K Klein M Suzuki H Takada T Stiburkova B. Familial early-onset hyperuricemia and gout associated with a newly identified dysfunctional variant in urate transporter ABCG2. Arthritis Res Ther. (2019) 21:219. doi: 10.1186/s13075-019-2007-7, PMID: 31661014 PMC6819377

[26] Rognvaldsdottir V Brychta RJ Hrafnkelsdottir SM Chen KY Arngrimsson SA Johannsson E. Less physical activity and more varied and disrupted sleep is associated with a less favorable metabolic profile in adolescents. PLoS One. (2020) 15:e0229114. doi: 10.1371/journal.pone.0229114, PMID: 32413039 PMC7228054

[27] Zhang L Zhang W Xiao C Wu X Cui H Yan P. Using human genetics to understand the epidemiological association between obesity, serum urate, and gout. Rheumatology (Oxford). (2023) 62:3280–90. doi: 10.1093/rheumatology/kead054, PMID: 36734534

[28] Zhao M Zhai H Li H Wei F Ma H Liu Y. Age-standardized incidence, prevalence, and mortality rates of autoimmune diseases in adolescents and young adults (15-39 years): an analysis based on the global burden of disease study 2021. BMC Public Health. (2024) 24:1800. doi: 10.1186/s12889-024-19290-3, PMID: 38970015 PMC11227207

[29] Zhao W Li X Shi R Zhu Y Wang Z Wang X. Global, regional and national burden of CKD in children and adolescents from 1990 to 2019. Nephrol Dial Transplant. (2024) 39:1268–78. doi: 10.1093/ndt/gfad269, PMID: 38130213

[30] Wang Y Charchar FJ. Establishment of sex difference in circulating uric acid is associated with higher testosterone and lower sex hormone-binding globulin in adolescent boys. Sci Rep. (2021) 11:17323. doi: 10.1038/s41598-021-96959-4, PMID: 34462530 PMC8405811

[31] Ling RE Liu F Lu XQ Wang W. Emerging issues in public health: a perspective on China's healthcare system. Public Health. (2011) 125:9–14. doi: 10.1016/j.puhe.2010.10.009, PMID: 21168175

[32] Wang Y Lim H. The global childhood obesity epidemic and the association between socio-economic status and childhood obesity. Int Rev Psychiatry. (2012) 24:176–88. doi: 10.3109/09540261.2012.688195, PMID: 22724639 PMC4561623

[33] Li T Zhang H Wu Q Guo S Hu W. Association between triglyceride glycemic index and gout in US adults. J Health Popul Nutr. (2024) 43:115. doi: 10.1186/s41043-024-00613-4, PMID: 39113110 PMC11308556

[34] Liu CS Li TC Lin CC. The epidemiology of hyperuricemia in children of Taiwan aborigines. J Rheumatol. (2003) 30:841–5. PMID: 12672209

[35] Tsai H Tantoh DM Hsiao C Zhong J Chen C Liaw Y. Risk of gout in Taiwan biobank participants pertaining to their sex and family history of gout among first-degree relatives. Clin Exp Med. (2023) 23:5315–25. doi: 10.1007/s10238-023-01167-1, PMID: 37668883

[36] Chang Y Lin C Liu T Huang C Chung C Chen Y. Polygenic risk score trend and new variants on chromosome 1 are associated with male gout in genome-wide association study. Arthritis Res Ther. (2022) 24:229. doi: 10.1186/s13075-022-02917-4, PMID: 36221101 PMC9552457

[37] Chen SY Shen ML. Juvenile gout in Taiwan associated with family history and overweight. J Rheumatol. (2007) 34:2308–11. PMID: 17937457

[38] Bui C Lin L Wu C Chiu Y Chiou H. Association between emotional eating and frequency of unhealthy food consumption among Taiwanese adolescents. Nutrients. (2021) 13:2739–54. doi: 10.3390/nu13082739, PMID: 34444899 PMC8401002

[39] Koto R Nakajima A Horiuchi H Yamanaka H. Serum uric acid control for prevention of gout flare in patients with asymptomatic hyperuricaemia: a retrospective cohort study of health insurance claims and medical check-up data in Japan. Ann Rheum Dis. (2021) 80:1483–90. doi: 10.1136/annrheumdis-2021-220439, PMID: 34158371 PMC8522452

[40] Yokose C McCormick N Lu N Joshi AD Curhan G Choi HK. Adherence to 2020 to 2025 dietary guidelines for Americans and the risk of new-onset female gout. JAMA Intern Med. (2022) 182:254–64. doi: 10.1001/jamainternmed.2021.7419, PMID: 35099520 PMC8804972

[41] Lee J. Influences of exercise interventions on overweight and obesity in children and adolescents. Public Health Nurs. (2021) 38:502–16. doi: 10.1111/phn.12862 33569831

[42] Yeo C Kaushal S Lim B Syn N Oo AM Rao J. Impact of bariatric surgery on serum uric acid levels and the incidence of gout-a meta-analysis. Obes Rev. (2019) 20:1759–70. doi: 10.1111/obr.12940, PMID: 31468681

[43] McCormick N Rai SK Lu N Yokose C Curhan GC Choi HK. Estimation of primary prevention of gout in men through modification of obesity and other key lifestyle factors. JAMA Netw Open. (2020) 3:e2027421. doi: 10.1001/jamanetworkopen.2020.27421, PMID: 33231639 PMC7686865

