# Supplementary material for: Trends and burden of gout among adolescents aged 10–24 years: insights from the global burden of disease study 2021
Source: Front Public Health. 2025 Jun 4;13:1526141. doi: 10.3389/fpubh.2025.1526141 (PMC12174453; doi:10.3389/fpubh.2025.1526141)
Supplement: Supplementary file 1 [file Data_Sheet_1.pdf]

Supplementary Figures

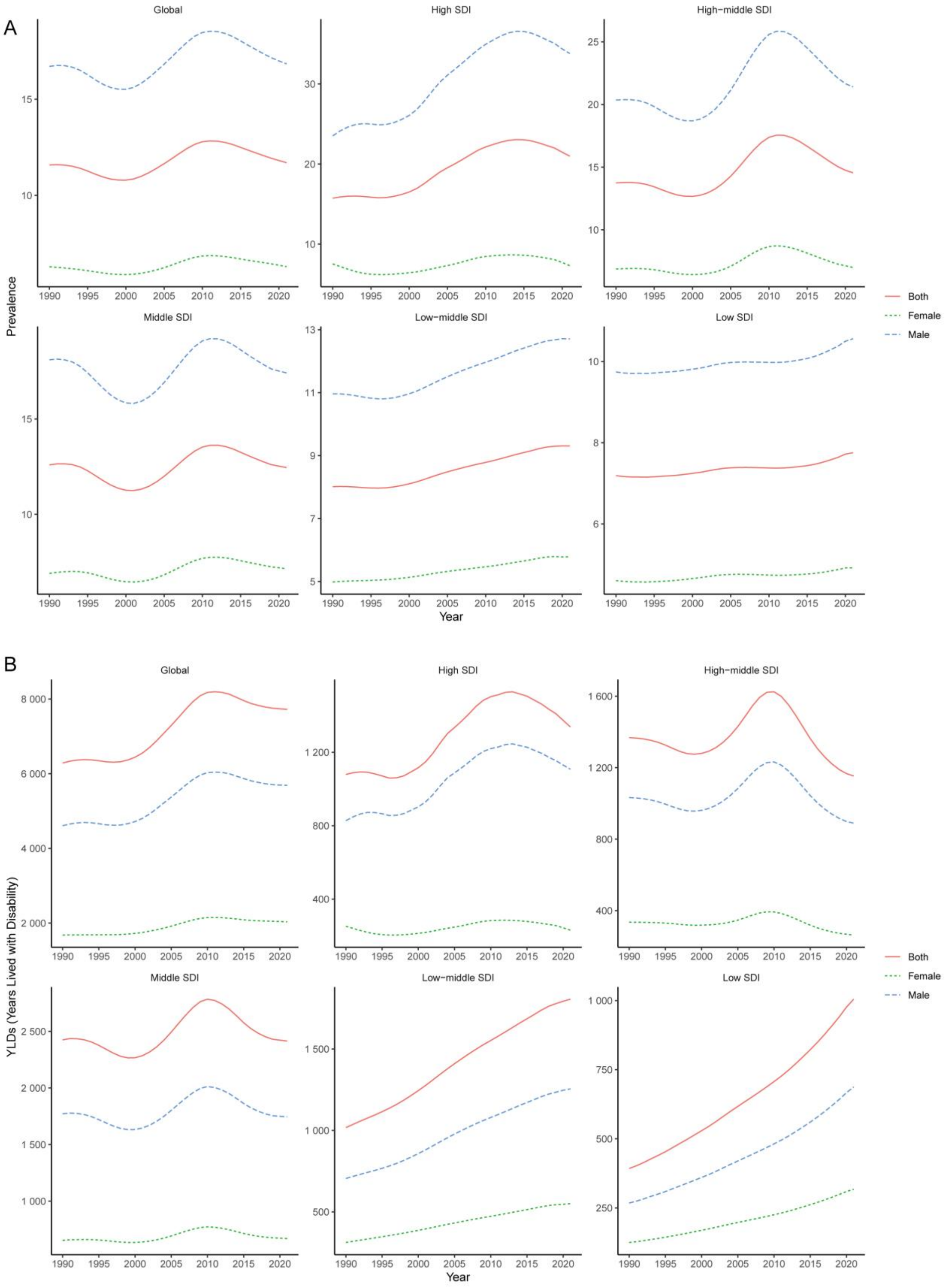

Supplementary Figure 1. **A** The age-standardized prevalence rate of gout among adolescents in high, high-middle, middle, low-middle, low SDI regions. **B** The YLD case of gout among adolescents in high,

high-middle, middle, low-middle, low SDI regions.

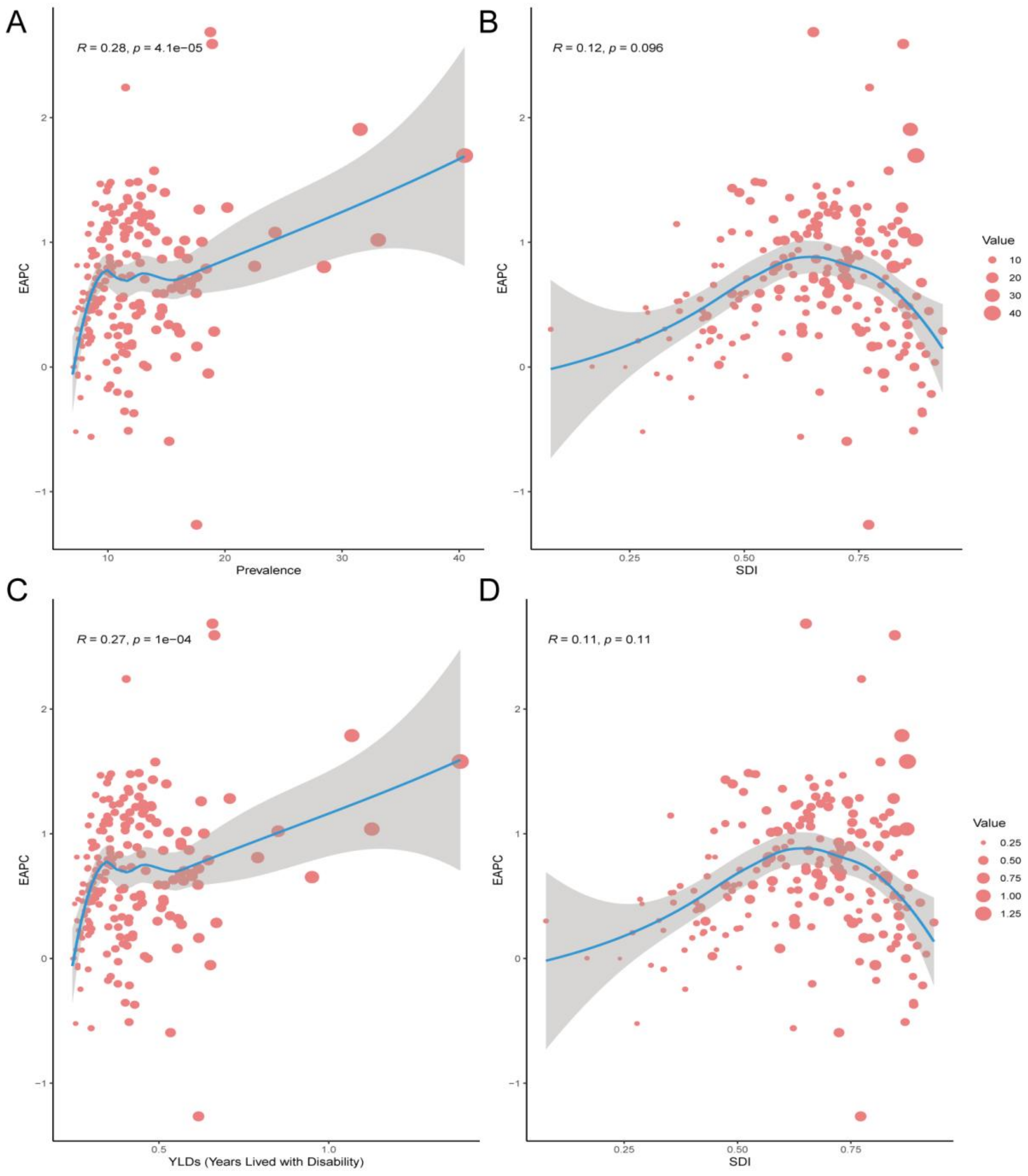

Supplementary Figure 2. **A**, **B** Analysis of correlation about prevalence, EAPC, SDI. **C**, **D** Analysis of correlation about YLD, EAPC, SDI.
