# Supplementary material for: Trends and burden of gout among adolescents aged 10–24 years: insights from the global burden of disease study 2021
Source: Front Public Health. 2025 Jun 4;13:1526141. doi: 10.3389/fpubh.2025.1526141 (PMC12174453; doi:10.3389/fpubh.2025.1526141)
Supplement: Supplementary file 2 [file Data_Sheet_2.pdf]

Supplementary Table 1. The prevalence of gout in 10-24 years old between 1990 and 2021 at the global and regional level

Location	Rate per 100 000 (95% UI)					
	1990		2021		1990-2021	
	prevalence cases	age-standardized prevalence rate	prevalence cases	age-standardized prevalence rate	cases change (%)	rate EAPC
Global	179232.87(93890.46-296682.18)	11.58(6.07-19.18)	220881.48(116542.40-364750.66)	11.70(6.17-19.32)	23.24(21.55-25.75)	0.381(0.205-0.558)
High	30812.09(16828.07-49416.57)	15.73(8.59-25.23)	38937.68(23429.04-59442.75)	20.98(12.63-32.03)	26.37(17.31-41.45)	1.515(1.279-1.751)
High middle	39001.10(20826.05-63222.87)	13.74(7.34-22.28)	32863.25(17858.84-53093.10)	14.55(7.91-23.50)	-15.74(-18.60--11.36)	0.813(0.486-1.141)
Middle SDI	69115.62(36456.30-114309.67)	12.59(6.64-20.83)	68816.86(35926.42-114707.00)	12.45(6.50-20.75)	-0.43(-2.87- 1.55)	0.299(0.087-0.512)
Low middle	28986.19(13616.28-49316.37)	8.01(3.76-13.63)	51458.53(25055.93-87155.58)	9.31(4.53-15.77)	77.53(72.63-85.42)	0.604(0.556-0.652)
Low SDI	11192.31(5069.50-19140.69)	7.19(3.26-12.30)	28646.15(13252.88-49063.81)	7.75(3.59-13.28)	155.95(149.70-165.33)	0.221(0.191-0.251)
Regions						
Andean Latin America	1064.04(473.38-1894.36)	8.64(3.85-15.39)	2121.59(1036.63-3597.83)	12.29(6.00-20.84)	99.39(81.00-129.45)	1.193(1.163-1.223)
Australasia	725.97(415.62-1131.86)	15.09(8.64-23.53)	1200.22(692.29-1858.61)	20.92(12.07-32.40)	65.33(47.23-94.46)	1.241(1.086-1.397)

Caribbean	979.66(428.40-1734.79)	9.17(4.01-16.25)	1262.18(595.84-2216.46)	11.14(5.26-19.57)	28.84(20.44-44.35)	0.715(0.585-0.845)
Central Asia	1661.96(771.29-2867.36)	8.38(3.89-14.46)	2092.48(1014.56-3549.40)	9.46(4.59-16.04)	25.90(18.82-35.62)	1.010(0.772-1.249)
Central Europe	2114.10(931.16-3624.75)	7.24(3.19-12.41)	1539.16(709.05-2616.55)	8.49(3.91-14.43)	-27.20(-30.81--21.40)	0.699(0.507-0.892)
Central Latin America	4805.54(2115.81-8560.33)	8.86(3.90-15.78)	7533.31(3558.23-13098.43)	11.58(5.47-20.14)	56.76(46.68-73.82)	0.420(0.225-0.616)
Central Sub-Saharan Africa	1329.51(621.82-2267.63)	7.68(3.59-13.10)	3469.02(1607.92-5937.34)	7.72(3.58-13.21)	160.92(137.20-183.59)	0.083(0.039-0.127)
East Asia	67057.85(38040.87-106768.66)	18.02(10.22-28.69)	43805.04(25473.02-68330.94)	18.03(10.48-28.12)	-34.68(-37.66 --31.09)	0.748(0.316-1.182)
Eastern Europe	3872.45(1779.44-6623.60)	8.20(3.77-14.02)	2753.28(1278.62-4722.96)	8.34(3.88-14.31)	-28.90(-32.44--24.14)	0.743(0.313-1.175)
Eastern Sub-Saharan Africa	4354.37(1944.53-7456.32)	7.02(3.13-12.02)	11346.73(5252.44-19518.76)	7.80(3.61-13.42)	160.58(151.42-174.41)	0.306(0.268-0.344)
High-income Asia Pacific	5012.64(2615.94-8237.87)	11.90(6.21-19.55)	3705.51(1950.23-5836.80)	14.19(7.47-22.36)	-26.08(-29.88--21.04)	0.253(0.133-0.373)
High-income North America	14507.15(8465.71-22737.25)	23.71(13.84-37.17)	22582.59(14470.23-32902.31)	31.68(20.30-46.16)	55.67(40.37-77.88)	1.821(1.469-2.174)
North	8957.28(4111.99-1	8.22(3.78-14.12)	16127.83(7839.54-26	9.94(4.83-16.62)	80.05(71.01-94.99)	0.806(0.668-0.944

Africa and Middle East	5381.69)		979.39)			)
Oceania	278.24(152.30-446.47)	13.30(7.28-21.34)	593.34(342.62-957.44)	14.71(8.50-23.74)	113.24(97.11-128.12)	0.340(0.282-0.399)
South Asia	25154.09(11407.19-42868.41)	7.52(3.41-12.82)	45241.38(20814.21-77474.69)	8.60(3.96-14.73)	79.86(74.47-87.20)	0.559(0.491-0.627)
Southeast Asia	17235.44(8993.51-27926.90)	11.62(6.06-18.82)	24810.47(13521.23-39625.88)	14.51(7.91-23.17)	43.95(37.45-54.28)	0.821(0.790-0.852)
Southern Latin America	1643.08(892.92-2674.62)	12.41(6.75-20.21)	2546.45(1451.21-4099.56)	16.60(9.46-26.73)	54.98(41.06-72.84)	0.929(0.883-0.975)
Southern Sub-Saharan Africa	1479.38(699.03-2521.24)	8.66(4.09-14.76)	2139.46(1043.78-3623.41)	9.81(4.78-16.61)	44.62(37.99-53.54)	0.651(0.523-0.779)
Tropical Latin America	3657.82(1564.65-6396.88)	7.64(3.27-13.37)	5344.28(2444.75-9054.48)	10.57(4.83-17.90)	46.11(33.77-68.01)	1.175(1.095-1.256)
Western Europe	8923.51(4308.39-14879.05)	10.86(5.24-18.10)	8047.72(4037.75-13201.80)	11.17(5.60-18.32)	-9.81(-14.36--3.21)	0.140(0.090-0.190)
Western Sub-Saharan Africa	4418.81(1993.91-7590.79)	7.38(3.33-12.68)	12619.46(5854.49-21606.07)	7.82(3.63-13.39)	185.59(177.69-197.76)	0.164(0.098-0.229)

Supplementary Table 2. The YLDs of gout in 10-24 years old between 1990 and 2021 at the global and regional level

Location	Rate per 100 000 (95% UI)					
	1990		2021		1990-2021	
	YLDs cases	age-standardized YLDs rate	YLDs cases	age-standardized YLDs rate	cases change	rate EAPC
Global	6287.14(2958.23-10998.57)	0.41(0.19-0.71)	7722.32(3648.10-13409.75)	0.41(0.19-0.71)	22.83(19.88-26.45)	0.368(0.192-0.544)
High	1079.41(528.60-1874.79)	0.55(0.27-0.96)	1338.01(691.11-2191.80)	0.72(0.37-1.18)	23.96(8.83-46.29)	1.443(1.210-1.677)
High middle	1368.32(665.93-2384.29)	0.48(0.23-0.84)	1153.13(565.05-1989.25)	0.51(0.25-0.88)	-15.73(-18.60--11.36)	0.813(0.486-1.141)
Middle SDI	2425.15(1166.83-4215.73)	0.44(0.21-0.77)	2414.66(1163.04-4220.35)	0.44(0.21-0.76)	-0.43(-2.87-1.55)	0.299(0.087-0.512)
Low middle	1017.10(436.81-1854.84)	0.28(0.12-0.51)	1805.84(794.88-3271.24)	0.33(0.14-0.59)	77.55(72.63-85.42)	0.604(0.556-0.652)
Low SDI	392.76(169.18-729.98)	0.25(0.11-0.47)	1005.11(441.51-1847.31)	0.27(0.12-0.50)	155.91(149.70-165.33)	0.221(0.191-0.250)
Regions						
Andean Latin America	37.32(16.41-68.76)	0.30(0.13-0.56)	74.45(33.75-135.50)	0.43(0.20-0.78)	99.50(81.00-129.45)	1.194(1.164-1.224)
Australasia	25.44(12.87-42.71)	0.53(0.27-0.89)	42.10(20.98-72.65)	0.73(0.37-1.27)	65.49(45.83-94.77)	1.233(1.080-1.387)
Caribbean	34.38(14.89-63.23)	0.32(0.14-0.59)	44.29(19.66-81.17)	0.39(0.17-0.72)	28.84(20.44-44.35)	0.715(0.585-0.845)
Central Asia	58.33(24.86-105.86)	0.29(0.13-0.53)	73.38(33.25-130.97)	0.33(0.15-0.59)	25.81(18.82-35.62)	1.009(0.770-1.248)

Central Europe	74.17(30.70-140.89)	0.25(0.11-0.48)	53.99(23.19-100.63)	0.30(0.13-0.55)	-27.20(-30.81--21.40)	0.699(0.507-0.892)
Central Latin America	168.69(71.93-318.42)	0.31(0.13-0.59)	264.47(118.96-504.11)	0.41(0.18-0.78)	56.78(46.68-73.82)	0.420(0.224-0.616)
Central Sub-Saharan Africa	46.66(20.14-86.09)	0.27(0.12-0.50)	121.77(53.19-225.44)	0.27(0.12-0.50)	160.97(137.20-183.59)	0.084(0.040-0.127)
East Asia	2352.80(1182.87-4053.47)	0.63(0.32-1.09)	1535.93(784.72-2601.88)	0.63(0.32-1.07)	-34.72(-37.94--30.98)	0.744(0.312-1.178)
Eastern Europe	135.90(57.21-249.75)	0.29(0.12-0.53)	96.61(42.32-176.55)	0.29(0.13-0.54)	-28.91(-32.44--24.14)	0.743(0.313-1.175)
Eastern Sub-Saharan Africa	152.83(65.24-284.55)	0.25(0.11-0.46)	398.17(172.05-725.39)	0.27(0.12-0.50)	160.53(151.42-174.41)	0.305(0.267-0.343)
High-income Asia Pacific	175.88(82.75-310.28)	0.42(0.20-0.74)	130.01(61.16-225.25)	0.50(0.23-0.86)	-26.08(-29.88--21.04)	0.252(0.132-0.372)
High-income North America	507.36(257.28-865.64)	0.83(0.42-1.42)	765.26(412.11-1319.17)	1.07(0.58-1.85)	50.83(20.11-90.30)	1.716(1.364-2.068)
North Africa and Middle East	314.29(138.27-572.19)	0.29(0.13-0.53)	565.79(261.82-1006.33)	0.35(0.16-0.62)	80.02(71.01-94.99)	0.806(0.668-0.944)
Oceania	9.76(4.86-16.97)	0.47(0.23-0.81)	20.82(10.29-35.98)	0.52(0.26-0.89)	113.20(97.11-128.12)	0.338(0.280-0.396)
South Asia	882.53(375.94-1637.69)	0.26(0.11-0.49)	1587.67(693.55-2935.13)	0.30(0.13-0.56)	79.90(74.47-87.20)	0.559(0.491-0.627)

Southeast Asia	604.71(281.20-1056.54)	0.41(0.19-0.71)	870.50(427.78-1494.40)	0.51(0.25-0.87)	43.95(37.45-54.28)	0.821(0.790-0.852)
Southern Latin America	57.65(27.99-99.90)	0.44(0.21-0.75)	89.33(44.18-154.44)	0.58(0.29-1.01)	54.95(41.06-72.84)	0.928(0.881-0.974)
Southern Sub-Saharan Africa	51.91(22.94-93.42)	0.30(0.13-0.55)	75.06(34.33-134.52)	0.34(0.16-0.62)	44.59(37.99-53.54)	0.651(0.523-0.778)
Tropical Latin America	128.32(52.66-243.13)	0.27(0.11-0.51)	187.51(83.79-338.21)	0.37(0.17-0.67)	46.12(33.77-68.01)	1.175(1.095-1.256)
Western Europe	313.13(141.20-566.08)	0.38(0.17-0.69)	282.40(125.17-501.55)	0.39(0.17-0.70)	-9.81(-14.36--3.21)	0.139(0.089-0.189)
Western Sub-Saharan Africa	155.07(66.28-286.59)	0.26(0.11-0.48)	442.78(193.55-810.21)	0.27(0.12-0.50)	185.54(177.69-197.76)	0.163(0.098-0.228)
